# Metabolomics, heart disease and aging

**DOI:** 10.18632/aging.202804

**Published:** 2021-03-10

**Authors:** Michela Piedepalumbo, Walter J. Koch, Claudio de Lucia

**Affiliations:** 1 Independent Researcher, Naples, Italy; 2 Center for Translational Medicine, Lewis Katz School of Medicine, Temple University, Philadelphia, PA 19140, USA

**Keywords:** aging, heart disease, metabolism, metabolomics, elderly

Recent advances in preventive and lifestyle medicine in conjunction with improvements in pharmacological and surgical treatments have prolonged the life expectancy with a significant increase in the number of patients 65 years of age and older [1]. However, many elderly patients are vulnerable or frail with multiple comorbidities [1,2].

Heart disease (including but not limited to coronary artery disease, myocardial ischemia and heart failure) and metabolic diseases (diabetes, obesity, dyslipidemia and metabolic syndrome - a cluster of different conditions) are common disorders in the elderly population and represent a major health problem worldwide causing high hospitalization and mortality as well as elevated costs [1]. Nevertheless, geriatric patients are usually under-represented in clinical trials and the pathophysiological mechanisms behind aging and age-associated disorders are not completely understood. Diabetes, obesity, dyslipidemia and insulin resistance are independent risk factors for cardiovascular diseases including micro-vascular dysfunction, atherosclerosis, acute coronary syndrome, and heart failure (HF). Metabolic disorders often occur together and are associated with worse prognostic outcomes in older patients with heart disease [2].

Elderly subjects show multi-organ impairment with metabolic dysfunction as well as a slow and progressive decline in adaptive homeostasis [3]. However, since older adults often display multiple comorbidities, it is difficult to understand the specific contribution of aging itself in addition to cardio-metabolic diseases to the overall metabolic dysregulation in the clinical setting.

Metabolomics is an emerging powerful technology that allows for the profiling of several metabolites in biofluids (e.g. whole blood, plasma, serum and urines) and tissues. Using a metabolomic focused approach not only allows identifying altered metabolites and circulating prognostic biomarkers during disease but also provides insight into the pathophysiological mechanisms involved.

Animal studies have confirmed that aging itself is characterized by impaired function and metabolic changes in different organs including liver, muscle and adipose tissue, resulting in a peculiar plasma metabolic profile (e.g. changes in fat and amino acid metabolism) [3,4]. This is in line with a recent study that involved serum metabolomics analysis of healthy elderly and young subjects: amino acids, glycoproteins and several lipids were impaired with age while amino acid and fatty acid metabolism pathways significantly contributed to the aging process [5] (Figure 1).

**Figure 1:**
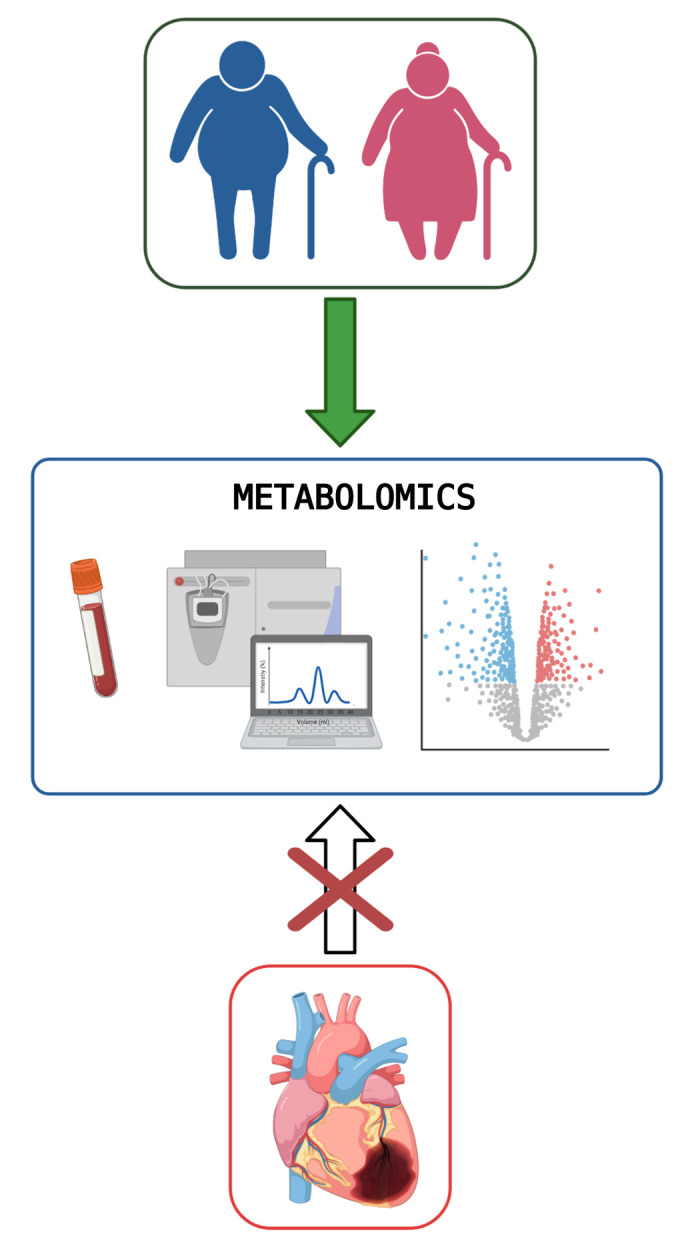
**Metabolomics, heart disease and aging.** Elderly subjects with heart disease show multi-organ impairment with significant metabolic alterations as confirmed by circulating metabolomics. Aging itself is characterized by metabolic changes in different organs including liver, muscle and adipose tissue resulting in a peculiar plasma metabolic profile (key changes are in fat and amino acid metabolism). Interestingly, the metabolic syndrome further affects the blood metabolome in the older subjects with alterations in amino acid metabolism being central features. Conversely, myocardial ischemic injury does not further alter the plasma metabolomic signature in old (and young) mice. Figure created with BioRender.com.

Plasma metabolomics studies have shown altered circulating metabolite levels in patients with HF (e.g. increased long-chain acylcarnitines and impaired levels of circulating metabolites involved in fatty acid oxidation) [6]. Of note, in these studies the average age of HF patients was > 60 years and specifically >65 years for patients with HF with reduced ejection fraction [6]. Importantly, the plasma metabolite profile was associated with survival rate and clinical outcomes in HF subjects [6]. However, the contribution of cardiovascular risk factors and myocardial ischemia/heart failure to the dysfunctional metabolomic profile displayed by patients with heart disease was not clarified in the human studies.

Our group has recently confirmed the circulating metabolic footprint of aging (altered amino acid, phospholipid and organic acid levels together with perturbed pathways involved in amino acid, glucid and nucleic acid metabolism as well as pyridoxal-5'-phosphate salvage pathway) and has demonstrated that myocardial ischemic injury does not affect the plasma metabolomic signature in either young or old mice [1]. This finding is of great interest as HF is a complex clinical syndrome, with impairment of several systems including the endocrine, musculoskeletal and renal systems. Our data suggest that other comorbidities (most probably metabolic disorders) may play a key role in the disarrangement of the metabolic profile during heart disease in the older adults

Roberts and collaborators performed a metabolomic study in serum samples from two ethnically distinct cohorts of elderly patients (from the United Stated and Japan) and evaluated the effect of the metabolic syndrome [7]. The authors found that the metabolic syndrome affected the metabolic profile in the older subjects with alterations in amino acid metabolism being central features. Interestingly, another study showed that overweight/obesity and diabetes were deleterious and associated with increased acceleration of metabolomic aging (defined as the difference, at a given age, between chronological and metabolomic age) [8].

Further studies will elucidate how different metabolic disorders affect tissue and circulating metabolome in older subjects with heart disease. Translational studies will be instrumental to comprehend the pathophysiological mechanisms involved and will clarify the specific contribution of hyperglycaemia, insulin resistance and dyslipemia to the metabolic disturbances occurring in elderly patients with HF.

## References

[1] de Lucia C, et al. Aging Cell. 2021; 20:e13284. 10.1111/acel.13284 33377274 PMC7811846

[2] Veronica G, Esther RR. Aging Dis. 2012; 3:269–79. 22724085 PMC3375083

[3] Srivastava S. Metabolites. 2019; 9:301 10.3390/metabo9120301 31847272 PMC6950098

[4] Houtkooper RH, et al. Sci Rep. 2011; 1:134. 10.1038/srep00134 22355651 PMC3216615

[5] Chen L, et al. J Proteome Res. 2020; 19:3264–75. 10.1021/acs.jproteome.0c00215 32434331

[6] McGarrah RW, et al. Circ Res. 2018; 122:1238–58. 10.1161/CIRCRESAHA.117.311002 29700070 PMC6029726

[7] Roberts JA, et al. Int J Mol Sci. 2020; 21:1249. 10.3390/ijms21041249 32070008 PMC7072861

[8] Robinson O, et al. Aging Cell. 2020; 19:e13149. 10.1111/acel.13149 32363781 PMC7294785

